# Autophagy genes AMBRA1 and ATG8 play key roles in midgut remodeling of the yellow fever mosquito, *Aedes aegypti*

**DOI:** 10.3389/finsc.2023.1113871

**Published:** 2023-01-16

**Authors:** Najla M. Albishi, Subba Reddy Palli

**Affiliations:** Department of Entomology, University of Kentucky, Lexington, KY, United States

**Keywords:** midgut, metamophosis, RNAi, ATG, AMBRA1, *ambra*

## Abstract

The function of two autophagy genes, an activating molecule BECN1 regulated autophagy (AMBRA1) and autophagy-related gene 8 (ATG8) in the midgut remodeling of *Aedes aegypti* was investigated. Real-time quantitative polymerase chain reaction (RT-qPCR) analysis of RNA samples collected from the last instar larvae and pupae showed that these two genes are predominantly expressed during the last 12 h and first 24 h of the last larval and pupal stages, respectively. Stable ecdysteroid analog induced and juvenile hormone (JH) analog suppressed these genes. RNA interference (RNAi) studies showed that the ecdysone-induced transcription factor E93 is required for the expression of these genes. JH-induced transcription factor krüppel homolog 1 (Kr-h1) suppressed the expression of these genes. RNAi-mediated silencing of *AMBRA1* and *ATG8* blocked midgut remodeling. Histological studies of midguts from insects at 48 h after ecdysis to the final larval stage and 12 h after ecdysis to the pupal stage showed that *ATG* gene knockdown blocked midgut remodeling. *AMBRA1* and *ATG8* double-stranded (dsRNA)-treated insects retained larval midgut cells and died during the pupal stage. Together, these results demonstrate that ecdysteroid induction of *ATG* genes initiates autophagy programmed cell death during midgut remodeling. JH inhibits midgut remodeling during metamorphosis by interfering with the expression of ATG genes.

## 1 Introduction

Metamorphosis is associated with tissue remodeling in holometabolous insects, including the yellow fever mosquito, *Aedes aegypti* (1). Molting and metamorphosis are regulated by juvenile hormone (JH) and 20-hydroxyecdysone (20E is the most active form of ecdysteroids). JH is an anti-metamorphosis hormone essential for preventing metamorphosis (2). Steroid-induced programmed cell death is involved in the degeneration of the larval midgut, salivary glands, fat body, and other tissues and has been extensively studied in holometabolous insects (3). Programmed cell death (PCD) plays a vital role in insect metamorphosis and development. Programmed cell death, which includes apoptosis and autophagy, has been described only in few insects (3). However, most of the research is focused on apoptosis. Autophagic programmed cell death is a cellular mechanism highly conserved from yeast to mammals and depends on the lysosomal degradation pathway for elimination of dysfunctional cellular components. In eukaryotic cells, autophagy is regulated by a series of autophagy-related proteins (ATG) that function in the cellular process of autophagy: induction, nucleation, expansion, and completion of the autophagosome, which is followed by lysosomal fusion (4). The protein activating molecule BECN1 regulated autophagy (AMBRA1) is a novel regulator of autophagy that interacts with Beclin-1 (interact with BCL-2 protein) and stimulates its binding to vacuolar protein sorting-associated protein 34 (Vps34), which plays a role in autophagosome formation during autophagy (5). Increasing evidence of a pivotal role of AMBRA1 protein in autophagy and apoptosis has been reported in vertebrate neurodevelopment in previous years (6). A mutation in the *AMBRA1* gene impairs the regulation of autophagy in mice and alters the balance between apoptotic cell death and proliferation and resulting in embryonic lethality (7). From yeast to mammals, two conjugation systems are involved in the autophagosome formation process: the formation of the ATG12-5-16 complex on the isolation membrane and the localization of ATG8-PE to the isolation membrane. ATG12 and ATG8, ubiquitin-like proteins, play crucial roles in phagophore expansion and the formation of autophagosomes, which also requires other ATG proteins such as ATG4, ATG3, ATG7, and ATG10 (4). The ubiquitin-like protein ATG8 promotes the expansion of the isolation membrane and autophagosome membrane formation. ATG8 remains attached to the autophagosome until it is trafficked to the lysosome, where ATG4 releases it after the autophagosome is fused with the lysosome to form the autolysosome (8). Because of these reasons, ATG8 protein has been utilized as a marker for autophagic activity and autophagosome formation (9).

In insects, autophagy is an integral part of developmental processes in the remodeling of larval tissues (10, 11). In the fruit fly, *Drosophila melanogaster*, autophagy, and apoptosis are involved in the degradation of larval salivary glands; the silencing of *ATG* genes impairs the elimination of this organ (10). Autophagy has been reported to be involved in larval midgut degradation. Larval midgut degradation was delayed by inhibition of autophagy signaling in the *ATG2* mutant larvae or knockdown of *ATG1* and *ATG18* by RNAi during metamorphosis. In contrast, the overexpression of *ATG1* triggers premature midgut degradation (12). In lepidopteran insects, the removal of the larval tissues requires both the canonical apoptosis machinery and autophagy detected in the midgut (13), silk glands (14), and fat body (15, 16) during metamorphosis. The function of ATG, the critical regulator protein during metamorphosis, is largely unknown in the *Ae. aegypti* midgut remodeling.

The induction of autophagy by 20E has been explored in the midgut and fat body (13, 17). The PCD is promoted by 20E in the absence of JH; the increased levels of 20E induce apoptosis and autophagy (16, 17). In *D. melanogaster*, E93 influences autophagy by regulating a subset of *ATG* (10, 18), and ecdysone-response genes (*BR-C*, *E74*, *HR3*, and *βftz-F1*). The Target-of-Rapamycin (TOR) negatively regulates autophagy in the *D. melanogaster* fat body during larval development, and 20E directly regulates autophagy by targeting the PI3K pathway (19, 20). Here, we have examined the function of *ATG* genes in the *Ae. aegypti* midgut remodeling. To learn insights into autophagy’s role in *Ae. aegypti* midgut remodeling, we studied two core autophagy genes, *AMBRA1* and *ATG8* and discovered that the interaction between JH, 20E and autophagy plays a crucial role in regulating autophagy-dependent midgut remodeling in *Ae. aegypti*.

## 2 Methods

### 2.1 Insect rearing and staging

*Ae. aegypti* mosquitoes from Liverpool IB12 (LVP-IB12) strain were maintained in the laboratory at 27 ± 1°C temperature and 70-80% relative humidity with a photoperiod of 16:8 light/dark cycle, as previously described (1). The developmental markers were used to identify the stages of mosquito larvae (21).

### 2.2 RT-qPCR

Total RNA was isolated and used to quantify mRNA levels using gene-specific primers (**Table S1**) and RT-qPCR as described previously (1). The *RPS7* gene (AAEL009496) was used as a reference gene for normalization, and the 2^−ΔΔCT^ method was used to calculate the relative mRNA levels.

### 2.3 RNAi-mediated knockdown of ATG genes

For dsRNA preparation, fragments of the target genes were amplified from genomic DNA using Taq Polymerase (Taq 2XMaster Mix, NEB), Double-stranded RNA synthesis and preparation of poly-L-lysine (PLL), epigallocatechin gallate (EGCG),and dsRNA nanoparticles were prepared as described previously (22). Diet pellets containing 50 µg of AMBRA1, ATG8, E93, Kr-h1, or GFP dsRNA were made by mixing the dsRNA/PLL/EGCG complexes with a Bovine liver powder diet and were fed to the early third instar larvae (15 larvae per pellet) daily until they pupated or died. The knockdown efficiency in the 4th instar larval stage was determined using RT-qPCR.

### 2.4 Histology studies

For midgut morphological analysis, midgut dissected from larvae at 48 h AEFL and pupae at 12 h AEPS were dissected, fixed, stained, and photographed as described previously (1). The midgut sections were cut, processed, and photographed.

Additional details on Methods used in these studies are included in the supplementary information.

## 3 Results

### 3.1 Expression of *AMBRA1* and *ATG8* during metamorphosis

**Figure 1:**
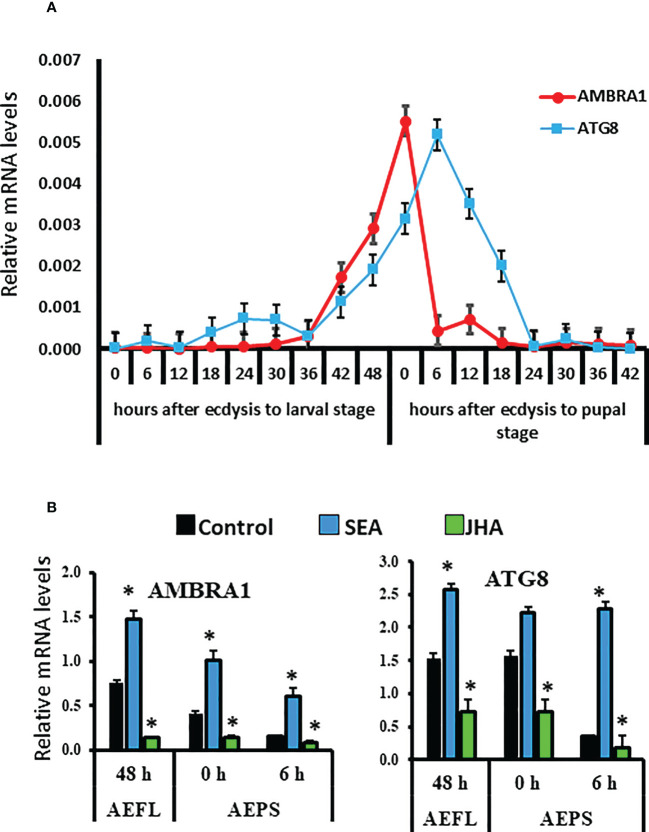
Developmental expression and hormone response of *AMBRA1* and *ATG8* genes during the final instar larval and pupal stages of *Aedes aegypti*. **(A)** Developmental expression of two autophagy genes *AMBRA1* and *ATG8* in the midgut of last instar larvae and pupae. Total RNA was isolated from midguts from staged insects. The cDNA and gene specific primers (**Table S1**) were used in RT-qPCR to determine relative mRNA levels of *AMBRA1* and *ATG8.* The *RPS7* gene was used as a reference gene for normalization, and the 2^−ΔΔCT^ method was used to calculate the relative mRNA levels. **(B)** Induction of *AMBRA1* and *ATG8* by stable ecdysteroid analog (SEA) and their suppression by JH analog (JHA). Relative mRNA levels are shown as Mean ± SE (n=4). The asterisks indicate significant difference at a p-value of <0.05.

RT-qPCR was used to determine mRNA levels of two autophagy-related genes, *AMBRA1* and *ATG8*, in the midguts collected at 6 h intervals during the larval-pupal metamorphosis. The results showed that *AMBRA1* mRNA levels increased at the end of the final instar larval stage and reached the maximum levels by 0 h after ecdysis into pupal stage (AEPS), then decreased by 6 h AEPS, and remained low during the rest of pupal stage (**Figure 1A**). In contrast, the *ATG8* mRNA levels increased beginning at 42 h after ecdysis to final instar larval stage and reached the maximum levels at 6 h AEPS. The mRNA levels then decreased to reach undetectable levels by 24 h AEPS. (**Figure 1A**). The mRNA levels of AMBRA*1* and *ATG8* were measured in different tissues, including the brain, midgut, fat body, and epidermis of the last instar larval and pupal stages. In general, higher levels of *AMBRA1* and *ATG8* mRNAs were detected in the midgut and fat body than in the other two tissues (**Figure 1S**). Expression of both *AMBRA1* and *ATG8* during the last 12 h of the last instar larval stage and early pupal stage and their expression in the midgut of larval and pupal stages suggest their involvement in midgut remodeling during the metamorphosis of *Ae. aegypti*.

### 3.2 Regulation of *ATG* genes expression by ecdysteroids and juvenile hormone

**Figure 2:**
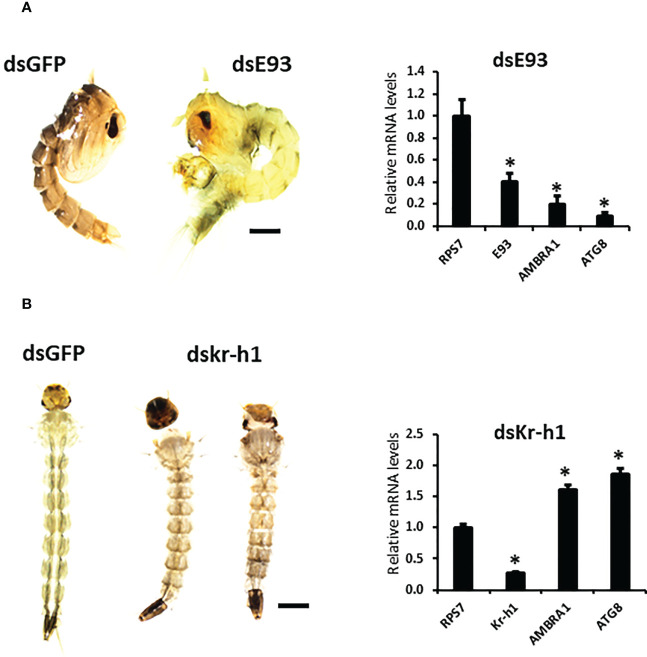
*D*ecreased expression of *ATG* genes in *E93* and *Kr-h1* knockdown *Aedes aegypti* larvae. **(A)** E93 is required for the 20E-dependent activation of ATG genes. Relative mRNA levels of *E93*, *AMBRA1*, and *ATG8* in dsE93 fed larvae. **(B)** Relative mRNA levels of *Kr-h1*, *AMBRA1*, and *ATG8* in the dsKr-h1 fed larvae. Data shown as Mean ± SE (n=4). The scale bar is 1000 µm. The asterisks indicate significant difference at a p-value of <0.05.

To study the effect of hormones on the midgut remodeling, the midguts were dissected from larvae at 48 h after ecdysis into the final instar larval stage (AEFL), and pupae at 0 and 6 h AEPS developed from larvae exposed to JH analog (JHA) methoprene, stable ecdysteroid analog (SEA) RH-102240 or the control larvae treated with DMSO. RT-qPCR analysis of RNA isolated from these midguts showed that E93 and USP-A mRNA levels increased in SEA-treated insects and Kr-h1 mRNA levels increased in JHA-treated insects compared to their levels in control insects, suggesting that hormone analogs are active and functioning as expected (**Figure 2S**). The mRNA levels of AMBRA1 and ATG8 are higher in SEA-treated insects and lower in JHA-treated insects compared to their levels in control insects (**Figure 1B**). Similar response to SEA and JHA was detected for other ATG genes studied (**Figure 2S**). To determine if E93 and Kr-h1 regulate the expression of *ATG* genes, *AMBRA1* and *ATG8* mRNA levels were determined in *Ae. aegypti* larvae fed on ds*Kr-h1* and ds*E93* nanoformulations. Due to the challenge of delivering naked dsRNA to *Ae. aegypti* larvae, diet pellets containing PLL/EGCG nanoformulated dsRNA were fed to early third instar larvae. The dsE93 treated larvae developed to the pupal stage, but the adult development was blocked and died during the pupal stage (**Figure 2A**). The dsKr-h1 treated larvae did not undergo metamorphosis and died during the last instar larval stage (**Figure 2B**). The *AMBRA1* and *ATG8* mRNA levels increased in insects fed on dsKr-h1 and decreased in insects fed on dsE93 (**Figures 2A, B**). These results showed the antagonistic effects of E93 and Kr-h1 on the expression of the *AMBRA1* and *ATG8* in *Ae. aegypti* suggesting that these two transcription factors regulate the expression of *AMBRA1* and *ATG8*.

### 3.3 Knockdown of *AMBRA1* and *ATG8* blocked midgut remodeling

**Figure 3:**
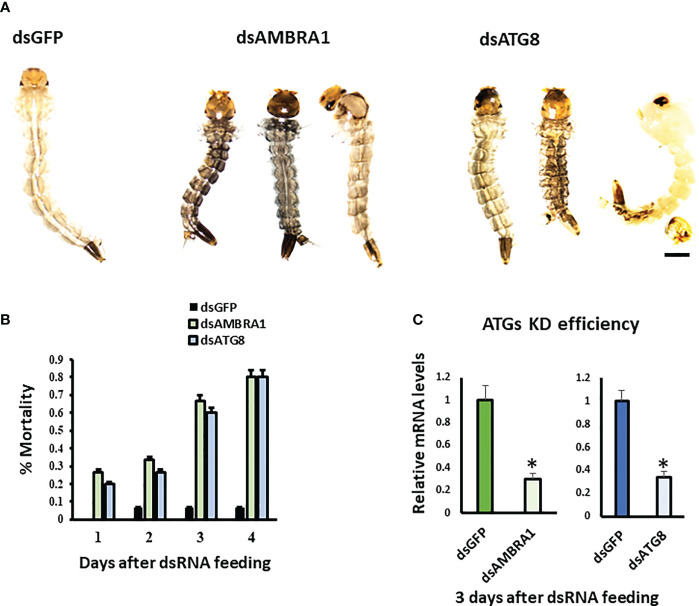
Phenotypes observed in final instar *Aedes aegypti*. larvae fed on ds*AMBRA1* and ds*ATG8* **(A)** The larval phenotypes observed after dsAMBRA1 and dsATG8 formulated with PLL/EGCG nanoparticles were fed to third instar larvae. The control larvae were fed on PLL/EGCG/dsGFP nanoparticles. The scale bar is 1000 µm. **(B)** ATG gene knockdown induces mortality of *Ae. aegypti* larvae. Percent mean mortality is shown (n= 30/treatment). **(C)** The Knockdown efficiency of dsAMBRA1 and dsATG8 in the last instar larva after three days of feeding. Relative mRNA levels are shown as Mean ± SE (n=4). The asterisks indicate significant difference at a p-value of <0.05.

To determine the function of *AMBRA1* and *ATG8*, RNAi was used to knockdown the expression of these genes. Diet pellets containing PLL/EGCG nanoformulations of dsAMBRA1, dsATG8, or dsGFP (as a control) were fed to early third instar larvae. Knockdown of *AMBRA1* and *ATG8* resulted in defects in larval growth and development (**Figure 3A**). The treated larvae were smaller and darker when compared to control larvae fed on dsGFP. In addition, feeding dsAMBRA1 or dsATG8 to the larvae for three days resulted in more than 60% mortality (**Figure 3B**). Compared to the control (dsGFP fed), the *AMBRA1* and *ATG8* mRNA levels decreased by 70% and 74%, respectively, in dsAMBRA1 and dsATG8 fed larvae (**Figure 3C**).

**Figure 4:**
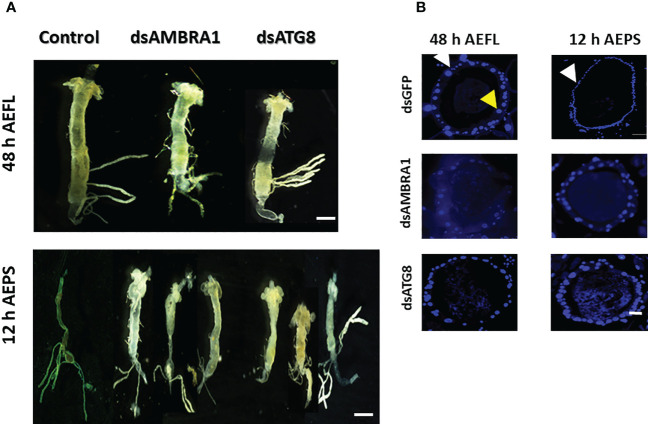
The effects of *AMBRA1* and *ATG8* knockdown on midgut remodeling in *Aedes aegypti*. **(A)** The larval midguts were dissected from larvae at 48 h AEFL and pupae at 12 h AEPS after feeding on dsAMBRA1 and dsATG8 and compared with the control larvae fed on dsGFP. The scale bar is 1000 µm. **(B)** Cross-sections of midguts of larvae at 48 h AEFL and pupae at 12 h AEPS developed from dsAMBRA1 and dsATG8 fed larvae compared with the control larvae. DAPI nuclear stain showed that the larval midgut epithelium contains two types of cells with small nuclei (white arrow) and large nuclei (yellow arrow). In the control pupa, the midguts showed only small nuclei-containing cells. The midguts from ATG gene knockdown insects showed both small and large nuclei. The scale bar is 50 μm.

The midgut remodeling begins within 36 h of ecdysis to the final larval instar (AEFL) and continues until 12 h AEPS (1). Therefore, we examined the knockdown efficiency and effects at two developmental time points: at 48 h AEFL and 12 h AEPS. Following dsRNA feeding, midguts were dissected from the larvae at 48 h AEFL and pupae at 12 h AEPS. Midguts from control larvae fed on GFP reached, and the gastric caeca began to degenerate at 48 AEFL (**Figure 4A**). By 12 h AEPS, the midguts in the control larvae were thin, and the gastric caeca degenerated (**Figure 4A**). The midguts dissected from larvae fed on dsAMBRA1 and dsATG8 did not show these changes in morphology. These midguts were long and wide with intact gastric caeca similar to those in larvae, which indicated that midgut remodeling was blocked in these insects (**Figure 4A**). The sections of the midguts were stained DAPI to detect the size of epithelial cell nuclei. The midguts from larvae 48 h AEFL (dsGFP-treated control) contained two types of epithelial cells: larval epithelial cells with large polyploid nuclei (indicated with a yellow arrow) and stem cells has small diploid nuclei (marked with a white arrow) (**Figure 4B**). The pupal midgut consisted mostly of new epithelial cells with small diploid nuclei, where the larval epithelial cells migrated toward the lumen and formed the meconium. Midguts dissected at 12 h AEPS of larvae fed on dsAMBR1 or dsATG8 showed mostly larval cells (large polyploid nuclei) and a few cells with small nuclei. These data suggest that there is a block in the degeneration of larval cells in insects fed on dsAMBR1 and dsATG8. Together, these results demonstrate that *AMBRA1* and *ATG8* are expressed in the midgut tissues and play an important role in PCD of the larval midgut in *Ae. aegypti*.

## 4 Discussion

The studies reported here revealed a critical role for ATG genes in the midgut remodeling of Ae*. Aegypti*. AMBRA1 is a novel regulator for phagophore nucleation, and the knockout of *AMBRA1* in mice resulted in autophagy inhibition and embryonic lethality (5–7). ATG8 is essential for autophagic vesicle formation and has been utilized as a marker for autophagic activity in previous studies (9). Therefore, *AMBRA1* and *ATG8* have been common targets in autophagy studies. Disruption of autophagy contributes to blocking cell death in *D. melanogaster* (12), *B. mori* (11), and *H. virescens* (23) midgut and other larval tissues in insects (15–17). Studies in *Galleria mellonella* indicated that the formation of autophagosomes in the fat body cells concomitantly with the upregulation of *ATG6* and *ATG8* genes were observed during the early pupal stage (24). In the mosquito *Aedes albopictus*, *ATG8* was found to be expressed across all developmental stages, and the *ATG8* mRNA levels are higher in female adults than in male adults (9). *Ae. aegypti* females require a cascade of autophagy cytoplasmic events for oocyte development (25). The involvement of AMBRA1 as a regulator of autophagy and development processes has also been reported in mouse embryos, and its loss leads to abnormal embryonic development. AMBRA1 also regulates neurogenesis and cancer in mammals (6), suggesting that the function of *ATG* genes may be conserved.

RNAi-mediated *AMBRA1* and *ATG8* knockdown caused defects in midgut remodeling and death during the pupal stage in *Ae. aegypti*. Several studies have shown that inhibiting autophagy genes has a significant impact on development. In *D. melanogaster*, the *ATG1* mutants die before pupation (10), and in *Bombyx*, knockdown of several *ATG* genes caused lethality during prepupal and pupal stages (16), suggesting autophagy is a key player in metamorphosis. Moreover, previous studies have demonstrated that autophagy promotes cell death in larval midgut tissues during metamorphosis. In *D. melanogaster*, knockdown of the *ATG* genes using RNAi delayed midgut degeneration, and the overexpression of *ATG1* resulted in accelerated degradation of midgut tissues and contributed to pupal death (12, 26). Additionally, ATG proteins also participate in midgut remodeling during the larval-pupal transition in *B. mori* (11). Knockdown of *ATG12* in *H. armigera* larvae delayed pupation and midgut PCD (13). These results support our finding that autophagy contributes to midgut remodeling in *Ae. aegypti*.

JHA suppressed the expression of *AMBRA1* and *ATG8* genes, while SEA upregulated their expression in the midgut. JHA repressed the expression of *ATG* genes in the 4th instar larval stage, resulting in a defect in programmed cell death, and midguts maintained their larval cells. JH has been shown to inhibit autophagy in the fat body of *Mamestra brassicae* during the last larval instar, and RNAi of the JH receptor, methoprene tolerant had a significant effect on autophagic activity in *B. mori* and causing lethality during the larval-pupal transition (3). Studies in *D. melanogaster* and *B. mori* reported that 20E promotes both apoptosis and autophagy gene expression during metamorphosis (13, 27). In *B. mori*, an increase in *ATG* gene expression was observed in the fat body of larvae immediately after injection with 20E. EcR response elements (EcRE) are present in the *ATG1* promoter, and deletion of EcRE inhibited 20E-induced autophagy in *B. mori* (16). Another study demonstrated that the expression of *ATG1* and *ATG8* were inhibited upon *EcR* RNAi, which implies that 20E/EcR complex is involved in the regulation of *ATG* genes in the fat body of *Ae. aegypti* (25). The detailed molecular mechanism by which JH suppresses *ATG* expression requires further investigation.

Furthermore, these studies showed that both E93 and Kr-h1 are involved in JH and 20E hierarchical network that regulates autophagy. E93 has been classified as an “early” response gene of the ecdysone signaling pathway (28). SEA treatment upregulated E93 and JHA treatment upregulated *Kr-h1* expression resulting in the suppression of ATG genes and blocking of PCD cascade in the midgut (**Figure 2**). It is also possible that the JH early response gene *Kr-h1* may influence the *E93* and *ATG* expression levels in *Ae. aegypti.* In *D. melanogaster*, *E93* is induced by 20E and suppressed by JH (29) and Kr-h1 directly represses *E93* expression (30). The JH-mediated suppression of *E93* expression through Met and Kr-h1 has also been found in several other insects (29, 31). High JH levels may prevent the expression of *E93* during larval-larval molting. Therefore, the inhibition of midgut remodeling in JHA-treated insects is likely caused by the lack of E93. Moreover, RNAi of *E93* significantly decreased the mRNA levels of the *AMBRA1* and *ATG8* (**Figure 2**).

RNAi experiments suggested that E93-mediated 20E signaling activates *ATG* gene expression in the midgut during metamorphosis, while JH suppresses autophagy induction through transcription factor Kr-h1. Previous studies showed that the induction of *E93* by 20E determines a PCD response (31). In *Ae. aegypti*, E93 regulates autophagic cell death and expression of *ATG* genes, which is similar to what was observed in the *D. melanogaster* salivary gland and midgut (18). In *B. mori* E93 induces the expression of *ATG1* to promote the larval-pupal metamorphosis, and silencing of *E93* by the RNAi disrupted the steroid-programmed cell death signaling, including caspase activity, autophagy, and cell dissociation during fat body remodeling (32). The E93 mediates 20E-induced autophagic cell death in the midgut and salivary glands, and a mutation in *E93* reduced the expression of the *ATG* genes in the salivary glands (10) and the midgut (33). In summary, both *B. mori* and *D. melanogaster* display increased expression of *ATG* genes promoted by E93, whereas JH suppresses the transcription of *E93*, indicating that JH plays a negative role in the induction of autophagy through interaction with 20E signals through Kr-h1. Further studies are required to uncover the effects of JH on the interaction of ATG, E93, and Kr-h1 proteins in midgut remodeling. Overall, these studies revealed an important role of autophagy genes in the degradation of larval midgut in *Ae. aegypti*. Knockdown of *AMBRA1* and *ATG8* led to a block in midgut remodeling and death during the pupal stage. Application of JHA to the 4th instar larvae induced *Kr-h1*, suppressed *E93 expression* and autophagy. These data suggest that the autophagy is induced by 20E and suppressed by JH working through Kr-h1, E93 and ATG genes.

## Data availability statement

The original contributions presented in the study are included in the article/**Supplementary Material**. Further inquiries can be directed to the corresponding author.

## Author contributions

NA and SP participated in the research design. NA conducted the experiments. NA and SP prepared the manuscript. All authors contributed to the article and approved the submitted version.
