# Supplementary material for: Autophagy genes AMBRA1 and ATG8 play key roles in midgut remodeling of the yellow fever mosquito, Aedes aegypti
Source: Front Insect Sci. 2023 Jan 16;3:1113871. doi: 10.3389/finsc.2023.1113871 (PMC10926384; doi:10.3389/finsc.2023.1113871)
Supplement: Supplementary file 1 [file DataSheet_1.pdf]

Supplementary Material

JHA, methoprene, suppresses autophagy in the midgut cells of the yellow fever mosquito, *Aedes aegypti*.

Najla M. Albishi[1,2], and Subba Reddy Palli[1*]

[1] Department of Entomology, University of Kentucky, Lexington, KY, 40546, USA.

[2]Affiliation, Department of Biological Sciences, King Faisal University, Al-Hassa, 31982, Saudi Arabia.

*** Correspondence:**
rpalli@uky.edu

Methods

Insect rearing and staging

Ae. aegypti mosquitoes from Liverpool IB12 (LVP-IB12) strain were maintained in the laboratory at 27±1°C temperature and 70-80% relative humidity with a photoperiod of 16:8 light/dark cycle, as previously described [1]. Mosquitoes at adult stages were fed sterile 10% sucrose solution, and females were blood-fed on defibrinated sheep blood served in a small cup covered with Parafilm to act as a feeding membrane. After blood-feeding, eggs were collected on filter paper. The larvae were grown in a plastic tray filled with distilled water supplemented with 60% Bovine liver powder. The developmental markers were used to identify the stages of mosquito larvae [2].

RT-qPCR

Total RNA was isolated from several tissues such as the fat body, midgut, head, and epidermis dissected from a pool of 10-15 staged larvae or pupae. RNA was extracted from the tissues using TRI reagent, and total RNA quantity and quality were determined using a NanoDrop-2000 spectrophotometer. First-strand complementary DNA (cDNA) was synthesized using two µg of total RNA, M-MLV reverse transcriptase kit (Invitrogen, USA), and oligo (dT) primers. Real-time quantitative PCR (RT-qPCR) was carried out on the StepOnePlus™ Real-Time PCR System (Carlsbad, CA) using SYBR Green Master (Bio-Rad). Four biological replicates were used for each treatment. PrimerQuest IDT (www.idtdna.com) was used to design primer pairs using the basic primer design rules (Table S1). The *RPS7* gene (AAEL009496) was used as a reference gene for normalization, and the $2^{-\Delta\Delta CT}$ method was used to calculate the relative mRNA levels.

RNAi-mediated knockdown of ATG genes

For dsRNA preparation, fragments of the target genes were amplified from genomic DNA using Taq Polymerase (Taq 2XMaster Mix, NEB), and specific primers for AMBRA1, ATG8, E93, and Kr-h1 were used for PCR amplification (Table S1). The dsRNA targeting the jellyfish green fluorescent

protein (GFP) was used as a control. The PCR products were used as templates for dsRNA synthesis using the MEGAscript T7 Transcription kit (Invitrogen, USA), following the manufacturer's instructions. To deliver dsRNA, anoparticles containing poly-L-lysine (PLL), epigallocatechin gallate (EGCG), and dsRNA as described previously [3]. Diet pellets containing 50 μg of AMBRA1, ATG8, E93, Kr-h1, or GFP dsRNA were made by mixing the dsRNA/PLL/EGCG complexes with a Bovine liver powder diet and were fed to the early third instar larvae (15 larvae per pellet) daily until they pupated or died. The knockdown efficiency in the 4th instar larval stage was determined using RT-qPCR.

In vivo hormone analog treatments

The JH analog, methoprene (an isopropyl (2E,4E)-(RS)-11-methoxy-3,7,11-trimethyldodecane-2,4-dienoate), was dissolved in Dimethyl sulfoxide (DMSO). Technical grade stable ecdysone analog SEA, RH-102240 (N-(1,1-dimethylethyl)-N'-(2-ethyl-3-methoxybenzoyl)-3,5-dimethylbenzohydrazide) was dissolved in ethanol at 1 μg/μl concentration. Four independent groups of 100 mosquitoes in each group were selected from the newly 4th instar larval stage. The final concentration of each hormone was set at 100 ng/ml. The control group received the same volume of the solvent. A sample of each group of insects (JHA, SEA, and DMSO) was collected and staged according to their developmental markers [2].

Histology studies

For midgut morphological analysis, larvae at 48 h AEFL and pupae at 12 h AEPS were dissected in 1×PBS. Midguts were fixed in 4% PFA (Paraformaldehyde) at 4°C. The midgut tissues were washed twice with 1×PBS and observed using a Nikon SMZ745T stereo microscope. To prepare sections, the staged larvae and pupae were collected and fixed at 4°C in 4% Paraformaldehyde (PFA) for 24 h. The tissues were dehydrated in graded ethanol series (25, 50, 70, 90, and 100% ethanol; 20 min/step), infiltrated, and embedded in melting paraffin wax at 60°C. Sections of 5 μm were cut using the microtome (Leica RM 2135, Germany). Tissue sections were rehydrated and mounted with EverBrite^{TM}Mounting Medium with DAPI (Biotium, Hayward, CA, 23002). The stained sections were

photographed with a photomicroscope (1×71 Olympus Inverted Research Microscope supplied with a reflected fluorescence system) and Magnifier software.

Table S1. List of primers used in this study.

Name	Primer Sequence 5' to 3'	
	Forward	Reverse
RPS7	ACCGCCGTCTACGATGCCA	ATGGTGGTCTGCTGGTTCTT
AMBRA1	GGTGGAACGATCCGTCTTTAG	GAGCACACCTGCTCGAAATA
ATG8	CCCGTGATTGTTGAGAAAGC	ATTGTTGCCGATGTTGGTGG
ATG1	GCTGAATTTCGTCATCGCCC	CCCGGATGCGAGTAGATTCA
ATG3	TACGTCGGGGAGGAAACTCT	GTCCCCGTCTTCCAGATTCC
ATG4	ATGGATTACATGTTGGATGCA	TTACGCGATGATTTCGAACTC
ATG7	CCAGTGAACGGAAGCAGCTA	CCAGTGAACGGAAGCAGCTA
ATG10	AGCCAACGTGTACCTACGAA	TTCACCACTCCTCCATCCG
ATG12	CAAACATTCGCCCCATCTCC	TCATCCCCAGGCTTGACTCT
ATG13	ATCCTCCAATGCGAACAACCTG	CTTGGGTGACTGTGATAGCCG
ATG14	TCGGCGACTAATGGAGCCTA	GGACAGGTGTCGAGAAGTCC
ATG16	AAGTGAACTGGAACGAGCAATC	CTTTACTTTCCTATCAGCACCACC
Kr-h1	TTCTCGCAACAACAGCAACATCCG	TCATCAGATCCATTGACGCTGGGT
E93	AGACCAAAGCGAGGCAAGTA	AGATGCCGCTCCTTAACCTT
USA-A	TACGTGACAACGTGTCCGAGCG	TGTATTCGTACCGCGTCCGTCG
dsAMBRA1	CGATGAAGAAGACAGCGGAATA	GAGCACACCTGCTCGAAATA
ATG8	TACGATGGGTTCGCTCTACT	CTCATACACCAGGCAGAGATTG
dsE93	TAATACGACTCACTATAGGATAGTAC GCAACGGTCACTAC	TAATACGACTCACTATAGGACTACG CTGGTGTTGAGAAT
dsKr-h1	TAATACGACTCACTATAGGGCGAACT TCCACCAGACCAATAA	TAATACGACTCACTATAGGGAGGTT CTCCTTGACGCTAAAC

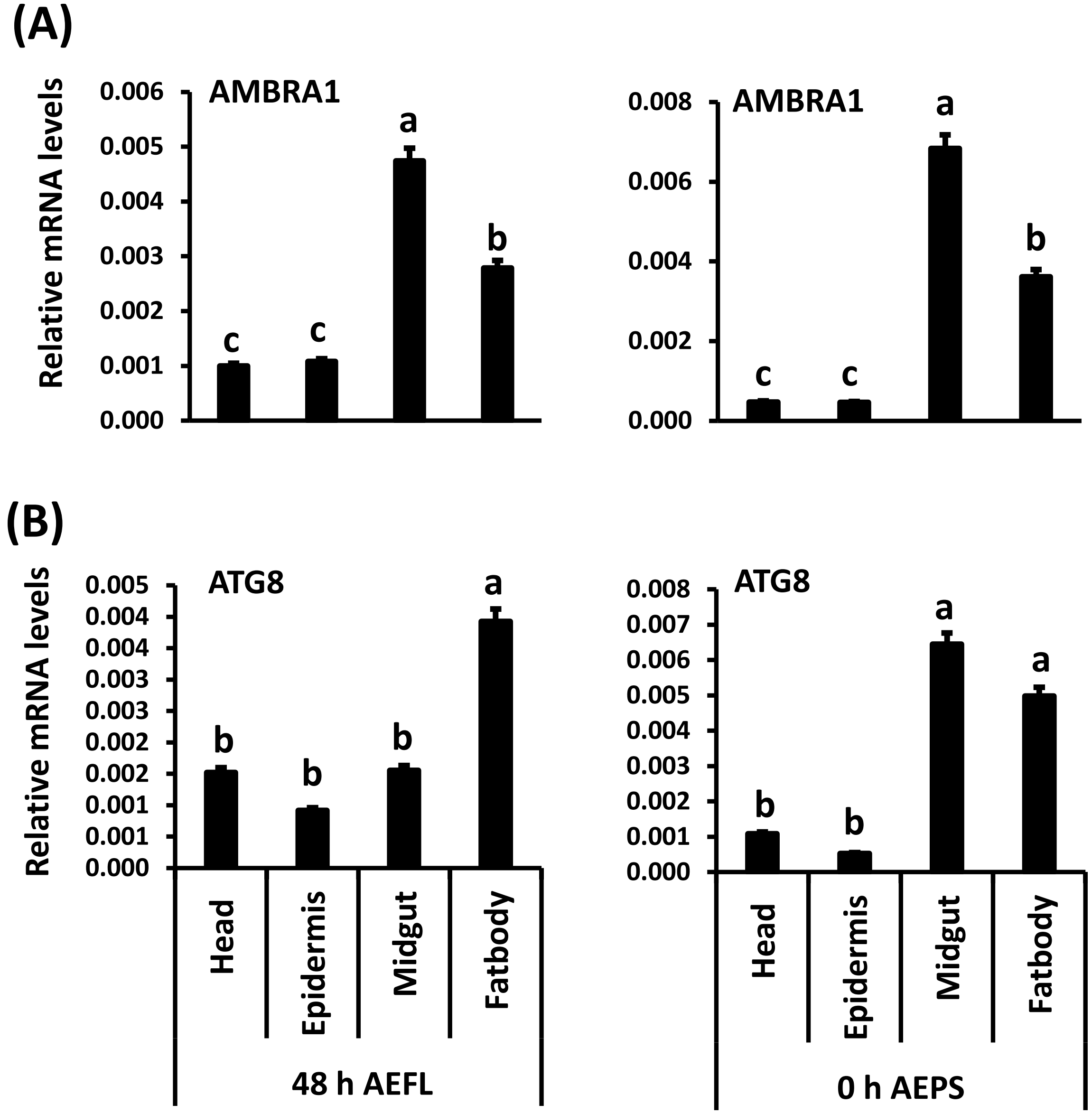

Figure 1S. Expression of *AMBRA1* and *ATG8* genes in the head, epidermis, midgut, and fat body tissues of 4th instar larvae and pupae. Total RNA was isolated from midguts from staged insects and used in RT-qPCR to determine relative mRNA levels of *AMBRA1* and *ATG8*. The *RPS7* gene was used as a reference gene for normalization, and the $2^{-\Delta\Delta CT}$ method was used to calculate the relative mRNA levels. The letters on the graphs indicate significant differences at a P-value of ≤ 0.05.

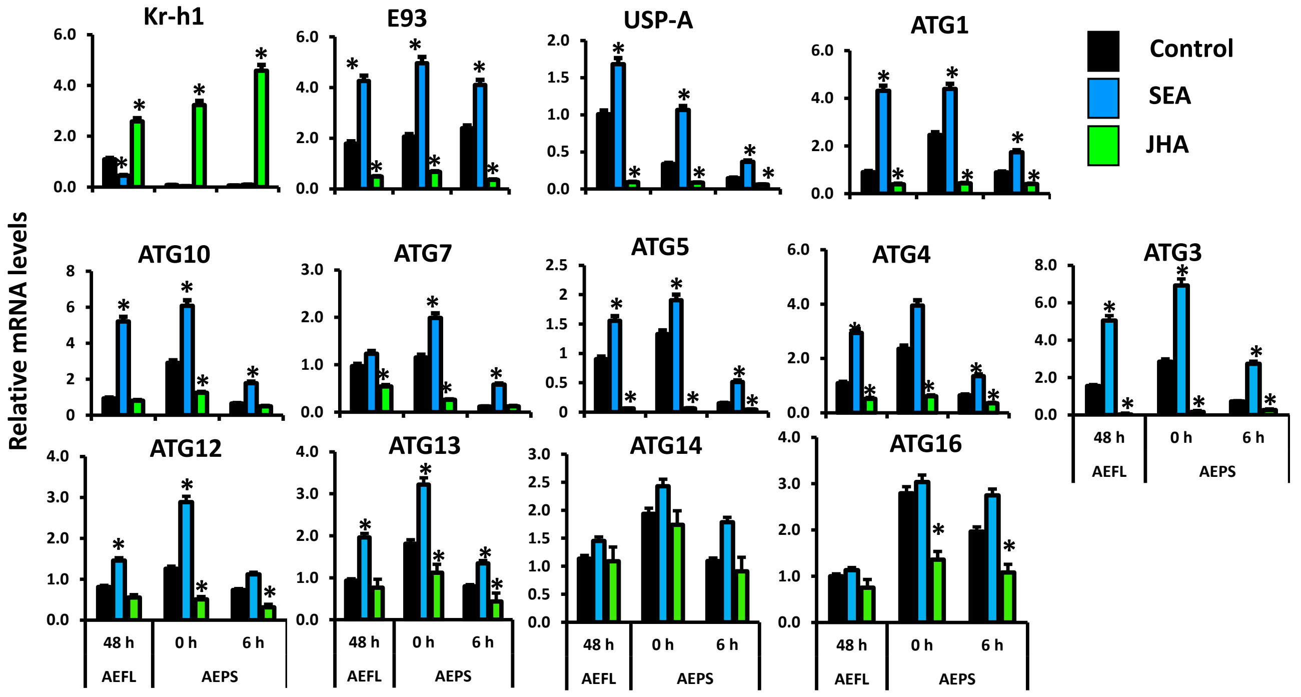

Figure 2S. The expression of ATG, JH, and 20E response genes during midgut metamorphosis. *Aedes aegypti* larvae were treated with SEA or JHA and the insects were collected at 48 h after ecdysis to the final larval stage (AEFL), 0 and 6 h after ecdysis to the pupal stage (AEPS). Total RNA was isolated from larval and pupal midguts and used in RT-qPCR to quantify the expression of ATG, JH, and 20E response genes. Relative expression was calculated based on the expression of the RPS7 gene as a reference. Data are shown as Mean ± SE (n=4).

References

[1] Y. Wu, R. Parthasarathy, H. Bai, and S.R. Palli, Mechanisms of midgut remodeling: juvenile hormone analog methoprene blocks midgut metamorphosis by modulating ecdysone action. Mechanisms of development 123 (2006) 530-547.

[2] J.T. Nishiura, Coordinated morphological changes in midgut, imaginal discs, and respiratory trumpets during metamorphosis of *Aedes aegypti* (Diptera: Culicidae). Annals of the Entomological Society of America 95 (2002) 498-504.

[3] R.K. Dhandapani, D. Gurusamy, and S.R. Palli, Development of Catechin, Poly-l-lysine, and Double-Stranded RNA Nanoparticles. ACS Applied BioMaterials 4 (2021) 4310-4318.
